# The influence of L4–S1 Dynesys® dynamic stabilization versus fusion on lumbar motion and its relationship with lumbar degeneration: a retrospective study

**DOI:** 10.1186/s13018-017-0597-9

**Published:** 2017-06-26

**Authors:** Chengmin Zhang, Liyuan Wang, Tianyong Hou, Lei Luo, Chen Zhao, Yibo Gan, Qiang Zhou, Pei Li

**Affiliations:** Department of Orthopedics, Southwest Hospital, Third Military Medical University, No. 29 GaoTanYan Street, Chongqing, 400038 China

**Keywords:** Posterior dynamic stabilization, L4–S1, Lumbar degenerative diseases, Range of motion, Adjacent segment degeneration

## Abstract

**Background:**

The aim of this study is to evaluate the efficacy of Dynesys® posterior dynamic stabilization (PDS) in the treatment of L4–S1 degenerative diseases and to assess the influence of postoperative motion on lumbar degeneration.

**Methods:**

Included in this retrospective study were patients with L4–S1 degenerative disease who underwent fusion or PDS from September 2010 to September 2014. Clinical outcomes were assessed by preoperative and postoperative visual analog scale (VAS) and Oswestry Disability Index (ODI). Preoperative and postoperative X-rays assessed range of motion (ROM) of the non-surgical and surgical levels and whole lumbar. MRI assessed degeneration of non-surgical levels.

**Results:**

A total of 56 consecutive patients were divided into two groups: group A, PDS, and group B, fusion. Patient demographics and baseline characteristics were similar in the two groups. In both groups, there was a significant difference between preoperative and postoperative VAS and ODI scores (*P* < 0.05). However, there was a significant difference in a 6-month follow-up ODI between the two groups (*P* < 0.05). X-rays showed PDS patients partially maintained surgical level ROM and non-surgical level ROM increased less than in the fusion group. MRI showed adjacent segment degeneration (ASD) in both groups, and patients whose preoperative L3–4 Pfirrmann classification was higher than grade 2 had more ASD than lower than grade 2.

**Conclusion:**

PDS can maintain surgical level ROM and had less influence on whole and non-surgical level ROM. Following PDS, patients recovered faster and had a better lumbar function. It may be a better choice for multi-level lumbar degenerative diseases.

## Background

Traditional posterior lumbar fusion is the primary procedure for multi-level lumbar degenerative disease [1]. Its purpose is to relieve clinical symptoms, and preserving lumbar motion is seldom considered. Recent biomechanical and clinical data show that range of motion (ROM) decreases at the fusion level, leading to ROM at the non-surgical level increase to compensate for the lost ROM, resulting in aggravation at the non-surgical level [2–7]. These conditions, in turn, can accelerate the degenerative process and may cause adjacent segment degeneration (ASD) and new symptoms [2–7].

Because of improved understanding of lumbar motion, surgeons now consider how to treat lumbar degenerative disease while maintaining ROM. New materials and techniques have led to the development of many non-fusion techniques and devices: nucleus replacement, artificial disc, interspinous process devices, and posterior dynamic stabilization. Lumbar non-fusion techniques treat the disease while maintaining ROM [8–10]. A systematic review comparing fusion with artificial disc replacement showed the lumbar artificial disc, which maintained ROM at the surgical level, had less effect on the adjacent level than fusion [11]. However, lumbar artificial discs have not been widely used because of a high failure rate. Dynesys® posterior dynamic stabilization (PDS) is one of the most widely used lumbar non-fusion techniques, with good clinical outcomes and fewer complications than other lumbar non-fusion techniques [12–15].

Few articles have reported on the efficacy of lumbar ROM and degeneration following decompression and different levels of PDS fixation [16–19]. A systematic review on the prevalence of ASD following spine surgery showed adjacent segments degenerate faster after multi-level fusion than single-level fusion [20]. The facet joints, ligaments, and spinal muscles have the most effect on spinal motion and maintaining spinal stability. Previous in vitro biomechanical studies and finite element studies usually only focused on the joints and ligaments, but not the spinal muscles. Therefore, radiological images can more comprehensively show the change in lumbar ROM.

**Fig. 1:**
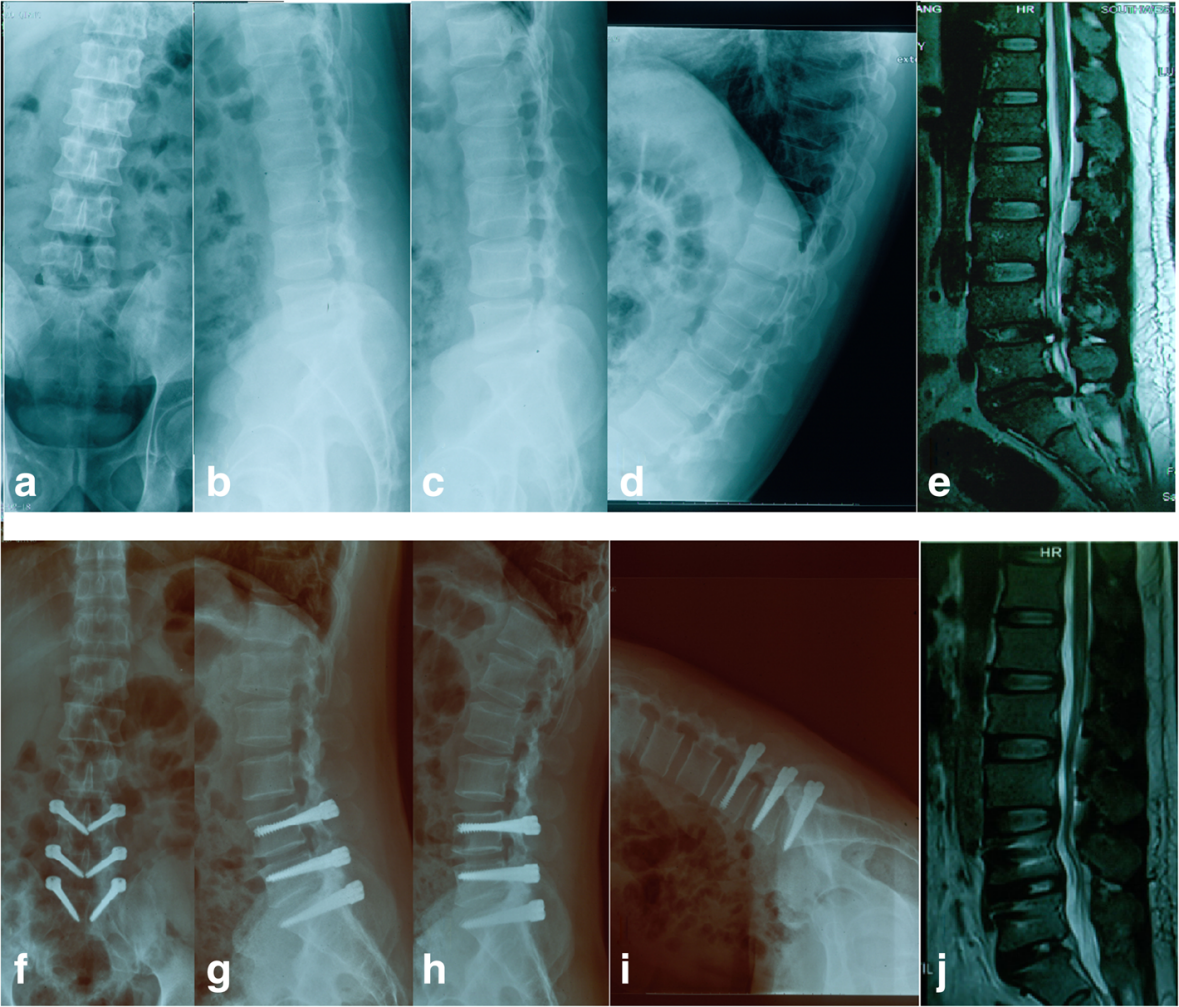
Thirty-three-year-old male with L4–5 and L5–S1 disc herniation who underwent L4–S1 PDS. **a**–**e** Preoperative radiological images. **f**–**j** Two years postoperative radiological images

Our study focuses on L4–5 and L5–S1, the most common degenerative lumbar levels. We evaluated the change in ROM before and after L4–S1 PDS (Fig. 1). Using patients who underwent L4–S1 fusion as a control group, we compared the two types of operations regarding their influence on ROM and the degeneration of the surgical levels, the non-surgical levels, and the whole lumbar spine, in order to clarify the different efficacy of lumbar degeneration following two operations.

## Methods

### Patients

This retrospective study included 69 consecutive patients with L4–S1 degenerative disease treated from September 2010 to September 2014. To compare clinical outcomes following L4–S1 Dynesys® PDS, traditional L4–S1 fusion was used as the control group.

Inclusion criteria were ages 18–65 years, clinical symptoms consistent with lumbar degenerative disease (radicular back or lower extremity pain and/or decreased muscular strength and/or abnormal sensation), radiographic evidence of L4–5 and L5–S1 disc degenerative disease (instability, grade I degenerative spondylolisthesis, stenosis, or disc herniation), history of L4–S1 decompression and fusion or Dynesys® stabilization, no symptoms caused by non-surgical levels, and at least had 2 years follow-up. Exclusion criteria were cauda equina syndrome, severe osteoporosis, dual-energy X-ray absorptiometry (DXA, previously DEXA) *T* score <−3.0, metabolic bone disease, ankylosing spondylitis, spinal deformity, tuberculosis, infection, tumor, or deformity; systemic infection such as acquired immune deficiency syndrome (AIDS) or active hepatitis; and mental illness, drug abuse, and/or alcoholism.

### Surgical technique

All operations were performed by one experienced surgical group (primary surgeon QZ). In the PDS group, a midline incision and Wiltse approach was used according to the manufacturer’s instructions (LIS, Top-Loading, & Zimmer® DTO®). Pedicle screws were inserted and the cord-rod constructs were installed. Dynesys® screws were fixed in such a way as to avoid influencing facet joint motion at the surgical and adjacent levels. Decompressions were performed at the levels indicated by imaging and patients’ symptoms. In the fusion group, all patients underwent L4–S1 transforaminal lumbar interbody fusion (TLIF).

### Clinical data

The visual analog scale (VAS) and Oswestry Disability Index (ODI) were performed preoperatively as well as postoperatively at 3 months, 1 year, and 2 years. X-rays and magnetic resonance imaging (MRI) were done preoperatively; both X-rays and MRI were done at 2 years postoperatively. The CT scan was done in fusion group at 6 months postoperatively, in order to confirm the interbody fused. If not, the CT scan would be done at 24 months.

### Radiological data

All radiological data was measured in the picture archiving and communication system (PACS) by two spinal surgeons. If there was dissent, the final decision was made by the corresponding author (QZ). To ensure consistency of the measurement results, the second measurement was done 4 weeks after the first one.

Measured preoperatively and at 2 years postoperatively were the lumbar sagittal Cobb angle; surgical segment sagittal Cobb angle; flexion, extension, and total ROM; surgical segment flexion, extension, and total ROM; non-surgical segment ROM; and non-surgical segment interspace disc height.

The following definitions were used:

- Sagittal Cobb angle: the angle between the superior endplates of L1 and S1 in the neutral lateral position.
- Surgical segment sagittal Cobb angle: the angle between the superior endplates of L4 and S1 in the neutral lateral position.
- Lumbar flexion, extension, and total ROM: First, *flexion and extension angles* were calculated by measuring the angles between the superior endplates of L1 and S1 in flexion and extension. *Flexion ROM*: The difference between the flexion and neutral angle. *Extension ROM*: The difference between the extension and neutral angle. *Total ROM*: The difference between the flexion and extension angle.
- Surgical segment flexion, extension, and total ROM: First, *surgical flexion and extension angles* were calculated by measuring the angles between the superior endplates of L4 and S1 in flexion and extension. *Surgical segment flexion ROM*: The difference between the surgical flexion and neutral angle. *Surgical segment extension ROM*: The difference between the surgical extension and neutral angle. *Surgical segment total ROM*: The difference between the surgical flexion and extension angle.
- Non-surgical segment ROM at L1–2, L2–3, and L3–4: First, *L3–4 flexion and extension angles* were calculated by measuring the angles between the superior endplate of L4 and the inferior endplate of L3 in flexion and extension. The difference between the flexion and extension angles was the *non-surgical segment L3–4 ROM*. The same procedure was followed for *L1–2 and L2–3*.
- Non-surgical segment interspace disc height: The average of the anterior and posterior L3–4 segment disc heights. The same procedure was followed for *L1–2 and L2–3*.
- Non-surgical segment disc degeneration: group 1, preoperative MRI Pfirrmann classification of L3–4 of ≤1.5; group 2, ≥2.
- Adjacent segment degeneration (ASD): Radiographic ASD (ASDeg) was defined as follows: (1) L3–4 disc height reduction more than 3 mm measured on the lateral X-ray; (2) L3 vertebral spillage of more than 3 mm compared with the preoperative on the lateral X-ray or the ROM of L3–4 segment was more than 15°; and (3) MRI evidence of aggravation of L3–4 degeneration, including L3–4 Pfirrmann grade increasing, disc herniation, or new stenosis. And the clinical ASD (ASDis) was defined as follows: (1) At least, the L3–4 segment had one of ASDeg and also had new clinical symptoms such as low back pain or lower extremity radiating pain.

### Statistical analysis

All data were analyzed using SPSS 19.0 statistical software (IBM-SPSS, Inc., Chicago, IL, USA). All data are presented as the mean ± standard deviation (SD). In both treatment groups, *t* test was used to assess patient age, body mass index (BMI), and follow-up time. Chi-square/Fisher’s test was used to assess gender. Independent sample *t* test was used to assess patient VAS and ODI. Independent sample *t* test was used to assess sagittal Cobb angle; surgical segment sagittal Cobb angle; flexion, extension, and total ROM; surgical segment flexion, extension, and total ROM; non-surgical segment ROM; and interspace disc height. A probability (*P*) value of <0.05 was considered statistically significant.

## Results

A total of 56 patients met inclusion and exclusion criteria. There were 27 patients in group A (PDS) and 29 patients in group B (fusion). Mean patient age, follow-up time, and BMI were similar in the two treatment groups. Mean patient age was 48.28 years in group A compared to 50.10 years in group B. Mean follow-up time was 28.78 months in group A compared to 29.90 months in group B. Mean BMI was 24.64 kg/m^2^ in group A compared to 25.03 kg/m^2^ in group B. Patient demographics and baseline characteristics for group A are shown in Table 1.

**Table 1:** Patient demographic data

	PDS	Fusion	*P*
Age	48.28 ± 2.44	50.10 ± 1.77	0.55^a^
Males/females	18/27	17/29	0.46^b^
Mean follow-up (months)	28.78 ± 0.61	29.90 ± 0.78	0.27^a^
Mean BMI (kg/m^2^)	24.64 ± 1.22	25.03 ± 1.64	0.28^a^
Operation level			
L4–S1	27	29	
Operation duration (h)	5.4 ± 1.2	6.8 ± 1.8	<0.01^a^
Blood loss (ml)	448.2 ± 219.6	602.2 ± 212.9	<0.01^a^

*BMI* body mass index

^a^Independent sample *t* test

^b^ *χ* ^2^ test

In both treatment groups, there was a significant difference between preoperative and postoperative VAS and ODI scores; patients in both groups experienced significant pain relief following treatment (*P* < 0.05). However, in both groups, there was no significant difference in pain relief between the postoperative and final follow-up. In addition, in the two treatment groups, there was no significant difference in preoperative, postoperative, or follow-up VAS values (*P* > 0.05). However, there was a significant difference in ODI values between the two groups at 6 months follow-up (*P* < 0.05), but no difference preoperatively or at 2 years follow-up (*P* > 0.05) (Table 2).

**Table 2:** Clinical outcomes

	Preoperative	6 months postoperative	24 months postoperative
Back pain VAS			
PDS	4.9 ± 1.3*	1.6 ± 0.4#	1.1 ± 0.6#
Fusion	5.7 ± 1.6*	2.0 ± 0.9#	1.4 ± 0.8#
Leg pain VAS			
PDS	5.5 ± 0.6*	1.2 ± 0.7#	0.5 ± 0.3#
Fusion	6.0 ± 0.8*	1.5 ± 0.9#	1.0 ± 0.5#
ODI			
PDS	52.6 ± 7.3*	17.1 ± 4.5#	12.2 ± 2.7#
Fusion	58.9 ± 9.6*	25.3 ± 5.6#	16.1 ± 4.3#

*P* was calculated with independent sample *t* test. * and # denote significant difference

*VAS* visual analog scale, *PDS* posterior dynamic stabilization, *ODI* Oswestry Disability Index

X-rays showed *whole lumbar ROM* decreased in the fusion group, especially *flexion ROM*. There was a significant difference between preoperative and postoperative ROM at 2 years follow-up (*P* < 0.05). However, the PDS group maintained ROM postoperatively. Comparing the two groups, there was a significant difference in postoperative *whole lumbar ROM* and *flexion ROM* (*P* < 0.05). Postoperative X-ray showed *surgical segment ROM* decreased in the fusion group, and there was a significant difference between preoperative and postoperative *surgical segment ROM* at 2 years follow-up (*P* < 0.05). However, the PDS group partly maintained *surgical segment ROM* postoperatively. Comparing the two groups, there was a significant difference in postoperative *surgical segment extension ROM*, *flexion ROM*, and *whole lumbar ROM* (*P* < 0.05) (Table 3).

**Table 3:** Summary of X-ray results

	PDS	Fusion	*P*
Lumbar sagittal Cobb angle			
Preoperative	34.53 ± 9.99	37.11 ± 7.85	0.29
Postoperative 2 years	35.60 ± 5.45	37.87 ± 4.96	0.11
Lumbar flexion ROM			
Preoperative	26.76 ± 12.50	30.11 ± 9.68*	0.27
Postoperative 2 years	24.69 ± 8.22	19.16 ± 4.20#	0.02
Lumbar extension ROM			
Preoperative	8.82 ± 4.50	7.98 ± 2.04	0.37
Postoperative 2 years	7.56 ± 4.26	7.06 ± 2.53	0.60
Lumbar total ROM			
Preoperative	35.04 ± 12.95	37.18 ± 10.15*	0.50
Postoperative 2 years	32.10 ± 8.40	26.23 ± 4.79#	0.02
Surgical segment sagittal Cobb angle			
Preoperative	23.37 ± 7.54*	24.79 ± 4.61	0.41
Postoperative 2 years	16.34 ± 4.80#	25.08 ± 3.18	<0.01
Surgical flexion ROM			
Preoperative	12.97 ± 6.06*	13.23 ± 3.98*	0.85
Postoperative 2 years	4.49 ± 2.39#	1.10 ± 0.65#	<0.01
Surgical extension ROM			
Preoperative	4.50 ± 2.67	3.99 ± 1.67*	0.40
Postoperative 2 years	3.14 ± 2.05	1.38 ± 0.90#	<0.01
Surgical total ROM			
Preoperative	16.96 ± 7.19*	17.32 ± 5.85*	0.83
Postoperative 2 years	7.10 ± 2.89#	2.38 ± 1.10#	<0.01

*P* was calculated with independent sample *t* test. Between preoperative and postoperative, * and # denote significant difference

*Non-surgical segment ROM* increased at all levels, and ROM at L3–4 mostly increased in both groups. There was a significant difference in ROM preoperatively and postoperatively in the fusion group (*P* < 0.05), but there was no difference in the PDS group (*P* > 0.05).

*Disc height* also decreased at L3–4 in both groups. There was a significant difference in disc height preoperatively and postoperatively in the fusion group (*P* < 0.05), but there was no difference in the PDS group (*P* > 0.05) (Table 4).

**Table 4:** Summary of non-surgical level X-ray results

	PDS	Fusion	*P*
L1–2 ROM			
Preoperative	5.41 ± 2.31	5.98 ± 1.86	0.32
Postoperative 2 years	6.91 ± 3.14	6.82 ± 3.74	0.93
L2–3 ROM			
Preoperative	7.03 ± 2.72	7.28 ± 1.75	0.70
Postoperative 2 years	7.53 ± 2.87	7.34 ± 2.50	0.80
L3–4 ROM			
Preoperative	7.51 ± 3.25	8.04 ± 2.90*	0.53
Postoperative 2 years	9.10 ± 3.03	11.06 ± 5.20#	<0.05
L1–2 disc height			
Preoperative	7.47 ± 1.54	7.52 ± 1.21	0.24
Postoperative 2 years	7.83 ± 1.56	7.75 ± 1.62	0.74
L2–3 disc height			
Preoperative	8.95 ± 1.29	8.79 ± 1.21	0.73
Postoperative 2 years	9.16 ± 1.24	8.94 ± 1.07	0.36
L3–4 disc height			
Preoperative	9.88 ± 2.09	9.92 ± 2.26*	0.46
Postoperative 2 years	9.34 ± 1.53	8.12 ± 1.38#	<0.05

*P* was calculated with independent sample *t* test. Between preoperative and postoperative, * and # denote significant difference

**Fig. 2:**
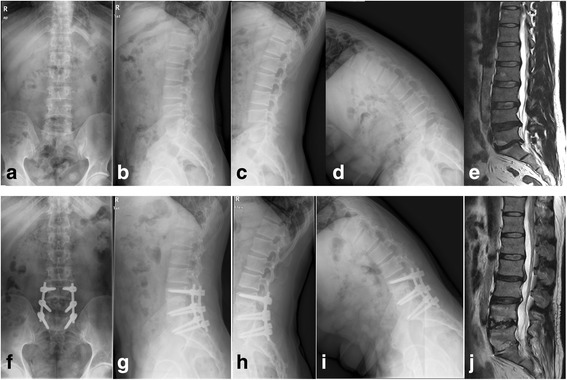
Forty-two-year-old male with L4–5 and L5–S1 disc herniation who underwent L4–S1 TLIF. **a**–**e** Preoperative radiological images. **f**–**j** Two years postoperative radiological images

MRI showed evidence of ASDeg in both groups. Patients whose preoperative L3–4 Pfirrmann classification was higher than grade 2 had more ASDeg (Tables 5 and 6). There were no neurovascular, spinal central cord, or nerve root injuries in either group, nor were there any screw-related complications or revision surgeries. One patient of fusion group had ASDis which clinical symptom was a recurrence of low back pain at 2 years follow-up, and MRI showed both L2–3 and L3–4 had degenerated. Clinical symptoms were relieved after conservative treatment (Fig. 2).

**Table 5:** The total ASD incident in both groups at 2 years postoperatively

	PDS	Fusion	Total
ASD	5	9	14
Negative	22	20	46
Total	27	29	56

*P* > 0.05

**Table 6:** Different Pfirrmann grade ASD incident at 2 years postoperatively

	PDS	Fusion	Total
Pfirrmann ≤1.5	1/11	2/12	3/23*
Pfirrmann ≥2	5/16	7/17	12/32
Total	6/27	9/29	15/56

* denotes significant difference

CT scan showed 54 levels of 26 patients of fusion group had been confirmed fused at 6 months postoperatively, and 2 patients in 4–5 levels had not fused, and 1 patient both in L4–5 and L5–S1 levels had not fused. At the 24-month postoperative, two patients who had single level did not complete fusion. So the fusion rate was 90% at 6 months postoperatively and 93% at 24 months.

## Discussion

Lumbar fusion surgery is the primary therapy for multi-level lumbar degenerative disease, but complications may occur, especially acceleration of ASD [20, 21]. Following improved knowledge of lumbar motion function, dynamic stabilization techniques developed quickly, and posterior dynamic stabilization has been accepted and used by more and more spine surgeons. The advantage of PDS is in maintaining *surgical segment ROM* with less effect on *whole lumbar ROM* than traditional fusion surgery.

Our results showed PDS can maintain 36% of *surgical level* (*L4–S1*) *ROM* 2 years postoperatively. *Flexion ROM* (at 33% preoperatively) decreased more than *extension ROM* (at 70% preoperatively). However, in the fusion group, there was almost no ROM at the surgical level following surgery. Because of the loss of ROM at the surgical level, *whole lumbar ROM* was also affected. *Whole lumbar ROM* was nearly the same preoperatively as in the PDS group, but was 70% in the fusion group; *flexion ROM*, which was 63% preoperatively, obviously decreased.

Yang et al. [22] compared dynamic stabilization and fusion for single and multi-level lumbar degenerative disease and found that dynamic stabilization maintained 36% of ROM at the surgical segment, but none in the fusion group. Yu et al. [18] reported that dynamic stabilization for multi-level lumbar degenerative disease maintained 48% of ROM at the surgical level. In contrast, *whole lumbar ROM* decreased 21% following dynamic stabilization and 40% following fusion. After fusion, ROM at the surgical level was almost lost in the fusion group. The authors concluded that dynamic stabilization maintains ROM better than fusion. While the non-surgical level partly compensated for ROM, *whole lumbar ROM* still decreased. However, because dynamic stabilization can partly maintain ROM at the surgical level, *whole lumbar ROM* is less affected. Yu et al. used the midline approach, in which the para-spinal muscles were dissected from the spinous processes and facet joint, which damaged muscle function. In our study, we used the Wiltse approach, in which para-spinal muscles were split to expose the screw insert points, which protected para-spinal muscle function in order to better maintain ROM.

Both types of surgery affect ROM at the surgical level, but compared to fusion surgery which involves rigid fixation and fusion, dynamic stabilization results in less loss of ROM. To accommodate ROM, non-surgical levels will compensate for the loss of ROM, especially at the adjacent levels. At postoperative follow-up of L4–S1 stabilization in both groups, ROM at the non-surgical levels increased. ROM at L3–4, which compensated most for lost ROM, increased more than ROM at L1–2 and L2–3. However, ROM at L3–4 in the PDS group increased 21% less than ROM in the fusion group, which increased 37%. ROM at L1–2 and L2–3 did not increase. Wang et al. [23] showed ROM at the first superior adjacent level increased after multi-level PDS and fusion operations, but adjacent level ROM in the PDS group increased less than in the fusion group. The authors concluded that dynamic stabilization had less influence on the non-surgical level. In summary, ROM at the non-surgical levels will increase to compensate for the loss of ROM after fusion surgery, and ROM at the first superior adjacent level increases more than at other levels.

Similar to extremity joints such as the knees, the spinal joints will degenerate faster if exercise frequency and intensity are increased. Consequently, degeneration of the discs and ligaments of the adjacent levels was accelerated. Our results showed disc heights at L3–4 decreased 18.1% in the fusion group and 5% in the PDS group; however, disc heights at L1–2 and L2–3 did not decrease after surgery. These results are consistent with the increase in ROM. It is known that decrease in disc space height is a signal of disc degeneration. We concluded that PDS maintained ROM at the surgical level; therefore, ROM at the non-surgical level increased less than that following fusion, which resulted in slower degeneration than that seen following fusion.

MRI showed that disc degeneration at L3–4 was worse postoperatively than preoperatively. In patients whose preoperative Pfirrmann classification was worse than grade 2, disc degeneration was faster than in the other group after both PDS and fusion surgery. In a meta-analysis by Xia et al. [20], 26.6% of the first adjacent level showed radiological degeneration and 8.5% showed new clinical symptoms.

There are some limitations of the Dynesys® dynamic stabilization system. In the PDS group, the postoperative surgical sagittal Cobb angle was just 70% of the preoperative angle, but in the fusion group, the surgical sagittal Cobb angle was almost the same as the preoperative angle. However, in both groups, the lumbar sagittal Cobb angle maintained well postoperatively. We believe the Dynesys® system can offer great support for the spinal posterior column, but there is a lack of support for the anterior and middle columns. After the discectomy, support for the anterior and middle columns declined. Therefore, the surgical level sagittal Cobb angle decreased. The length of spacer should be measured precisely during surgery, because a long spacer may cause surgical level kyphosis.

## Conclusion

In conclusion, our results showed PDS can maintain surgical level ROM and had less influence on whole lumbar and non-surgical level ROM. The patients recovered faster and had a better lumbar function. For multi-level lumbar degenerative diseases, PDS could reduce trauma and decrease fusion segments. It may be a better choice in the clinic.

## Abbreviations

AIDS: Acquired immune deficiency syndrome
ASD: Adjacent segment degeneration
DXA: Dual-energy X-ray absorptiometry
MRI: Magnetic resonance imaging
ODI: Oswestry Disability Index
PACS: Picture archiving and communication system
PDS: Posterior dynamic stabilization
ROM: Range of motion
TLIF: Transforaminal lumbar interbody fusion
VAS: Visual analog scale
